# Metallo-Drugs in Cancer Therapy: Past, Present and Future

**DOI:** 10.3390/molecules27196485

**Published:** 2022-10-01

**Authors:** Roxana Liana Lucaciu, Adriana Corina Hangan, Bogdan Sevastre, Luminița Simona Oprean

**Affiliations:** 1 Department of Pharmaceutical Biochemistry and Clinical Laboratory, Faculty of Pharmacy, “Iuliu-Hațieganu” University of Medicine and Pharmacy, 400012 Cluj-Napoca, Romania or; 2 Department of Inorganic Chemistry, Faculty of Pharmacy, “Iuliu-Hațieganu” University of Medicine and Pharmacy, 400012 Cluj-Napoca, Romania; 3 Clinic Department, Faculty of Veterinary Madicine, University of Agricultural Science and Veterinary Medicine, 400372 Cluj-Napoca, Romania

**Keywords:** cancer, metal complexes, platinum, copper, ruthenium

## Abstract

Cancer treatments which include conventional chemotherapy have not proven very successful in curing human malignancies. The failures of these treatment modalities include inherent resistance, systemic toxicity and severe side effects. Out of 50% patients administrated to chemotherapy, only 5% survive. For these reasons, the identification of new drug designs and therapeutic strategies that could target cancer cells while leaving normal cells unaffected still continues to be a challenge. Despite advances that have led to the development of new therapies, treatment options are still limited for many types of cancers. This review provides an overview of platinum, copper and ruthenium metal based anticancer drugs in clinical trials and in vitro/in vivo studies. Presumably, copper and ruthenium complexes have greater potential than Pt(II) complexes, showing reduced toxicity, a new mechanism of action, a different spectrum of activity and the possibility of non-cross-resistance. We focus the discussion towards past, present and future aspects.

## 1. Introduction

Cancer is a frequently lethal ilness caused by an abnormal cell growth with the ability to invade and spread throughout the organism; thus, it has the greatest incidence and mortality rate worldwide [1]. In the year 2020, the US was anticipated to witness 606,520 deaths and 1.8 million new cases [2]. About 4.5 million premature cancer-caused deaths were reported worldwide in 2016 [3]. Based on current data, the International Agency for Research on Cancer (IARC) forecasts that around 13 million cancer-related deaths will occur by 2030 [4].

There are hundreds different types of cancer affecting various organs, tissues, and cells (i.e., breast, bone, blood, colon, liver, lung, etc.) in the form of carcinomas, lymphomas, sarcomas, or leukemia, each one requiring specific treatment [5]. The most common clinical approaches for cancer treatment are chemotherapy, radiation therapy, surgery, hormone therapy, and targeted therapy with anticancer drugs [6].

The need for new and better pharmacological therapies for the treatment of cancer still exists. While there are succesful specific treatment frameworks against some types of cancer, such as anti-hormonal therapy in breast cancer [7], or monoclonal antibodies targeting aberrant receptors, the intrinsic heterogeneity found in cancer forces the use of highly toxic chemotherapeutic regimes [8].

The development of effective antitumor drugs with high selectivity and low toxicity is currently a major challenge for the scientific community. Indeed, the wide range of adverse effects resulting from cancer therapy have an impact on therapeutic adherence and general quality of life for patients and their families [9].

Cisplatin efficacy in the treatment of various cancers places coordinative chemistry among viable antitumor design alternatives. Although highly efficacious, treatment with cisplatin is still limited by side effects, inherited resistance, or acquired resistance, which has only partially been eliminated by the introduction of new Pt(II) drugs [10].

Other metal complexes containing ions such as copper, gold and zinc chelating agents have received great interest as anticancer agents [11,12,13]. Recently, the chemistry of ruthenium compounds has been intensively analyzed due to the interest in providing an alternative to cisplatin, because of their promising cytotoxic and potential anticancer properties [14,15,16]. 

Recently, there has been growing demand for metal-based compounds in the treatment of cancer. This may be due to the level of in vitro cytotoxic effects exhibited by recently synthesized metal-based compounds and to the fact that ligand substitution and the modification of existing chemical structures have led to the synthesis of a wide range of metal-based compounds, some of which have demonstrated an enhanced cytotoxic and pharmacokinetic profile [17].

The objective of this review is to provide an overview of previous studies on platinum, copper and ruthenium metalo-drugs in cancer therapy, focusing on past, present and future aspects.

## 2. Chemical and Biochemical Properties of Metallo-Drugs

The therapeutic potential of metal complexes in cancer therapy has attracted a lot of interest, mainly because metals exhibit unique characteristics, such as redox activity, variable coordination modes and reactivity towards the organic substrate. These properties have become an attractive probe in the design of metal complexes that selectively bind to the biomolecular target with a resultant alteration in the cellular mechanism of proliferation.

Several metal-based compounds have been synthesized with promising anticancer properties, some of which are already in use in clinical practice for diagnosis and treatment, while some are undergoing clinical trials [17].

### 2.1. Platinum Compounds

**Figure 1:**
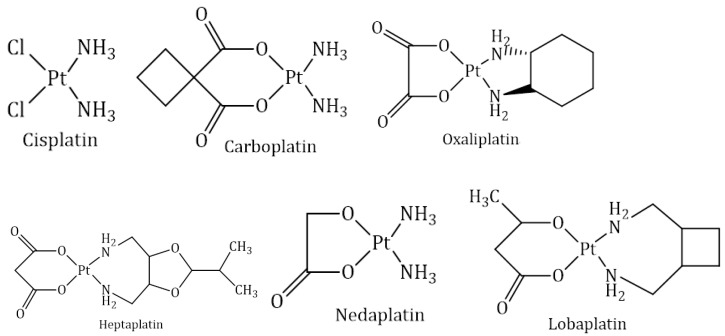
Chemical structures of cisplatin and its derivatives.

Platinum drugs attracted attention in cancer treatment once the antineoplastic activity of cisplatin was discovered in the 1960s. Cisplatin was the first metal-based anticancer drug introduced into clinical use. Since then, more platinum derivatives have been synthesised and tested against cancer cells; however, only a few have reached clinical trials. Nowadays, cisplatin and its derivatives are among the most frequently applied antitumor drugs in treating lung, colon, ovary, testicles, bladder, cervix and many more cancers. Cisplatin, carboplatin and oxaliplatin are used as worldwide anticancer drugs, while heptaplatin, nedaplatin and lobaplatin are used as regional anticancer drugs. Their chemical structures are shown in Figure 1 [18,19].

The platinum compounds currently used in therapy have common structural characteristics, namely, they are neutral compounds, with planar-square geometry and cis isomerism; they contain two ligands, ammonia molecules or other structures containing -NH_2_ groups (they do not leave the coordination sphere once the compound enters the tumor cell non-leaving groups) and they contain two anionic ligands that leave the coordination sphere (leaving groups) as a result of the intracellular activation process.

Nevertheless, cisplatin-based therapy has several disadvantages: it is highly toxic and cancer cells can acquire resistance relatively fast. Among the most negative side effects are neurotoxicity, nephrotoxicity and ototoxicity. To overcome these pharmacological limitations, new cisplatin derivatives are considered [19].

Cisplatin (1978):

Structurally, cisplatin has an arrangement whereby platinum ion is bounded to two chloride ions and two amine groups aligned in a square (Figure 1). Amine groups act as the carrier ligands, while chlorides act as the leaving groups. The arrangement of chloride ion is next to each other in cisplatin, which is biologically essential. Interestingly, transplatin, the trans isomer of cisplatin, does not exhibit anticancer activity [18].

The generalized mechanism of action for cisplatin and its derivatives involves four key steps: (1) cellular uptake, (2) aquation/activation, (3) DNA binding and (4) the cellular processing of DNA lesions, leading to apoptosis [20]. The two pathways by which this molecule is most likely to be taken up are passive diffusion through the plasma membrane and active transport, mediated by membrane proteins [21]. Cisplatin becomes activated intracellularly via hydrolysis reactions, where one of the two chloride leaving groups is replaced by a water molecule. Activated aqua platinum species react with nucleophilic sites in DNA, preferentially the nitrogen on position 7 of guanine (G), and form either monofunctional (via one leaving group) or bifunctional adducts (via two leaving groups). Although both inter- and intrastrand cross-links are possible, 1,2-interstrand d(GpG) cross-links (60–65% of all adducts) and d(ApG) cross-links (20–25% of all adducts) are the major products [22]. After the formation of DNA-platinum adducts in the nucleus, the consequent DNA damage is critical to cisplatin cytotoxicity, with several molecular mechanisms. For example, DNA damage initiates the release of cytochrome-c under the regulation of Bcl-2 family proteins, which activates procaspase-9, and then, forms an active apoptosome complex; DNA damage induces the activation of p53 protein, which could regulate cell death by counteracting the anti-apoptotic function of B-cell lymphoma-extra-large (Bcl-xL), the degradation of flice-like inhibitory protein, etc. In addition to DNA damage, it was reported cisplatin could induce reactive oxygen species to trigger cell death [19,23,24].

Cisplatin is accepted worldwide and is generally is used in the treatment of many types of cancer: ovarian, testicular, cervical, bladder, head, neck and lung (particularly the small cell type) [18].

Unfurtunatelly, cisplatin treatment produces several toxicities such as nephrotoxicity, hepatotoxicity, cardiotoxicity, myelosuppression, ototoxicity, gastrotoxicity, allergic reactions and some reproductive toxicities [18,25].

The fact that cisplatin occurs in the process of cell multiplication explains its more pronounced activity against tumor cells, with an accelerated rate of division, compared to that against healthy cells. This fact also explains the toxic effects that this compound has on the hematoforming marrow [18,26].

Mechanism of cisplatin resistance:

Cispaltin resistance happens once there is an insufficient amount of platinum ions to reach the target DNA or an insufficient number of Pt-DNA adducts generated to induce cell death [27]. 

For small molecular cisplatin, copper transporter-1 (CTR1), as the major plasma-membrane transporter, plays a substantial role in cisplatin influx. It was reported that the loss of CTR1 led to a 2- to 3-fold increase in drug resistance. In addition, efflux proteins such as ATP7A and ATP7B induce a decrease in intracellular cisplatin. Intracellular cisplatin exposure would trigger the rapid trafficking of ATP7A/B in the trans-Golgi towards cell periphery; during this process, ATP7A/B sequester cisplatin and consequently mediate the efflux of cisplatin [28]. Furthermore, once it has formed the Pt(GS)2 complex with glutathione (GSH), the cisplatin can also be eliminated by a multidrug resistance protein. There is still some cisplatin left inside the cancer cells, which could interact with intracellular thiol, amino, hydroxyl, etc. For example, the thiol-containing molecules, including glutathione/glutathione-S-transferase and metallothioneins, could inactivate and detoxify cytoplasmic cisplatin, leading to cisplatin resistance [29]. Even after Pt-DNA adducts are formed, cellular survival may still occur via several pathways, including DNA repair, the removal of these adducts and tolerance mechanisms, and eventually induce platinum resistance. Nucleotide-excision repair, mismatch repair, base-excision repair and double-strand-break repair are four major DNA repair pathways to remove cisplatin-induced DNA damage. Among them, as the main DNA repair pathway, nucleotide-excision repair involves the recognition and incision of DNA damage, followed by DNA synthesis to replace the excised fragments. Additionally, the core incision reaction requires the protein factors, including ERCC1-XPF, XPA, RPA, XPC-HR23B, TFIIH, XPG, etc. Additionally, mismatch repair is another important pathway to correct single base mispairs or looped intermediates through the identification and excision of the mismatch, and subsequent re-synthesis of the excised strand [22,24].

Carboplatin (1986):

Structurally, carboplatin it is diammine(1,1-cyclobutane dicarboxylato)platinum(II) (Figure 1). Carboplatin was developed in order to reduce the dose-limiting toxicity of cisplatin. It is obtained by replacing two chloride ions of cisplatin with a cyclobutane dicarboxylate ligand. It shows good aqueous solubility and high stability, resulting minimal side effects [18].

The mechanism of action of carboplatin is very similar to that of cisplatin; however, it has lower reactivity due to the bidentate ligand that replaces the chloride anions and that leaves the coordination sphere with a lower speed [24]. As a result of reduced reactivity, the nephrotoxicity, neurotoxicity and ototoxicity after carboplatin treatment are much less pronounced. Additionally, nausea and vomiting are less severe side effects and can be controlled easily. Due to its reduced toxicity profile, carboplatin is suitable for more aggressive high-dose chemotherapy. The myelosuppressive effect is considered to be one of the major drawbacks associated with carboplatin, with thrombocytopenia being more severe than neutropenia and anemia [18]. 

Carboplatin is accepted worldwide and generally is used in the treatment of ovarian cancer and of small cells lung cancer. It almost replaced cisplatin in combination regimens with paclitaxel for treatment of ovarian cancer [18,30]. The single intermittent bolus or short infusion shedule are more practical than the protracted infusion of cisplatin. Additionally, although carboplatin is more expensive than cispatin, the complete cost of the treatment is cheaper [31].

As in the case of cisplatin, tumor resistance to carboplatin poses a major clinical problem. The mechanisms underlying carboplatin resistance are generally similar to the mechanisms of cisplatin resistance. Unfortunately, cross-resistance ocurs to both platinum drugs [24].

Oxaliplatin (1996)

Oxaliplatin is a platinum complex with a 1,2-diaminocyclohexane ligand and oxalate as a leaving group (Figure 1) and was developed to overcome resistance against cisplatin and carboplatin. Oxaliplatin was initially launched in France in 1996 and was formally available in the countries of Europe in 1999 and in the US in 2002 [32]. It is licensed to be used as a combination therapy with other chemotherapeutic agents in the management of colon cancer and non-small-cell lung cancer [17,33]. In combination with 5-fluorouracil and folinate, oxaliplatin is used as an efficient treatment of adjuvant and metastatic colorectal cancer and is intrinsically insensitive to cisplatin. The bidentate oxalate significantly reduces the reactivity of oxaliplatin and thereby limits the toxic side effects to peripheral sensory neuropathy [34]. The diaminocyclohexane ligand is more lipophilic, increasing the passive uptake of oxaliplatin compared to cisplatin and carboplatin. Higher lipophilicity may also be a reason why oxaliplatin also employs some different routes of cellular entry to cisplatin and carboplatin. The organic cation transporters OCT1 and OCT2 have been implicated in mediating oxaliplatin uptake, as their overexpression significantly increases the cellular accumulation of oxaliplatin, but not cisplatin or carboplatin. Colorectal cancer cells overexpress organic cation transporters, which may explain the efficacy of oxaliplatin in this particular type of cancer [35]. The clinical relevance of these transporters remains, however, unclear. Regarding copper transporter-1 (CTR1), evidence that the transporter is involved in oxaliplatin uptake is not very strong, as in the case of cisplatin; nevertheless, the acquisition of oxaliplatin resistance was reported to be accompanied by CTR1 downregulation. Additionally, reduced expression of the β1-subunit of Na^+^, K^+^-ATPase was found in some oxaliplatin-resistant cells. Copper efflux transporters appear to play an important role in oxaliplatin sensitivity, too. Low levels of ATP7B in colorectal cancer patients were associated with a favorable outcome [36]. Similar to cisplatin, oxaliplatin mainly forms crosslinks on the adjacent guanine bases or between guanine and adenine, but to a lower extent. However, oxaliplatin-DNA adducts are more efficient in the inhibition of DNA synthesis. Due to the bulkier diaminocyclohexane ligand, oxaliplatin induces different conformational distortion on DNA. The bulkiness and lipophilicity of diaminocyclohexane are considered responsible for the differential processing of oxaliplatin-DNA adducts. The latter are not recognized by mismatch repair proteins. Interestingly, it does not lead to decreased cytotoxicity but makes oxaliplatin antitumor-activity mismatch repair proteins independent. Additionally, an increase in replicative bypass, i.e., DNA synthesis bypassing platinum-DNA adducts, was reported not to correlate with cytotoxicity of oxaliplatin. All these factors result in different activity spectra of oxaliplatin compared to cisplatin or carboplatin [24].

The diaminocyclohexane ligand plays a major role in cytotoxicity and protects oxaliplatin against cross-resistance with cisplatin [32]. Major side effects of oxaliplatin include nausea, numbness, low blood cell counts, allergic reactions and diarrhea [37]. It has a better safety profile than cisplatin, and is used for patients who cannot tolerate cisplatin [32].

Nedaplatin (1995)

Structurally, nedaplatin is a diammine-glycolatoplatinum compound (Figure 1) and is significantly more soluble in water compared to cisplatin. Like cisplatin, nedaplatin also has two amines: the carrier ligands and a glycolate containing a five-membered ring which is bonded to the platinum ion. It interacts with the nucleophilic groups of DNA, causing cell apoptosis [18].

It was approved only in Japan to treat head, neck, testicular, lung, esophageal, ovarian and cervical cancers [31,38]. 

A randomised clinical trial compared nedaplatin to cisplatin, both in combination with vindesine. Nedaplatin showed no advantage over cisplatin in objective response and overall survival, but was less toxic. More thrombocytopenia was observed, but there was less leucopenia, nephrotoxicity and gastrointestinal toxicity [31]. The drug is said to have a better safety profile than cisplatin and similar efficacy [17,18,39]. Compared to carboplatin, it is more toxic. Unfortunately, nedaplatin is cross-resistant with cisplatin [31].

Heptaplatin (1999)

Structurally, heptaplatin consists of malonate as a chelating leaving group and of 2-(1-methylethyl)-1,3-dioxolane-4, 5-dimethanamine as a chelating group (Figure 1). It was demonstrated to be stable in solution. Heptaplatin is currently applied in the Republic of Korea for the treatment of gastric cancer. Its antitumoral mechanism of action is assumed to be similar to that of cisplatin [18]. Heptaplatin was demonstrated to have fewer and milder side effects than cisplatin and to retain cytotoxic activity in cisplatin-resistant cell lines. A Phase III study showed that heptaplatin’s combination with 5-fluorouracil is comparable to cisplatin/5-fluorouracil regimen with less severe hematological side effects [34]. Studies regarding the use of heptaplatin as monotherapy or in combination with other antitumor agents continue [18,24,40].

Lobaplatin (2010)

Structurally, lobaplatin is represented as 1,2-diammino-l-methyl-cyclobutane-platinum(II)-lactate (Figure 1). Lobaplatin is approved in China for the therapy of metastatic breast cancer, chronic myelogenous leukemia and small cell lung cancer [34]. 

The antitumor activities of this compound span through the human lung, and ovarian and gastric cancer xenografts [32]. A phase I clinical trial of dose escalation of lobaplatin in combination with fixed-dose docetaxel in the treatment of human solid tumor was established. In this study, the maximum tolerable dose of lobaplatin when combined with docetaxel for the treatment of solid tumor, known to have progressed after chemotherapy, was established. Positive results from the phase I trial prompted the researchers to recommend the same dosage for the phase II clinical trial [18,41].

It has non-cross-resistance to cisplatin, particularly human sensitive cancer cells [17,21]. Concerning toxicity, its presence reduced nephrotoxicity, neurotoxicity and ototoxicity, but the hematotoxic effects are significant. Its dose-limiting toxicity is thrombocytopenia [34].

### 2.2. Copper Compounds 

The development of cisplatin as one of the most powerful anticancer drugs has opened new fields in the design of less toxic and more effective chemotherapy drugs based on the use of essential metals. It has been proposed that endogenous metals might be less toxic to normal cells than platinum, silver, or gold. In this regard, copper-based complexes present an attractive chemotherapy drug option [42,43].

The physiological concentration of copper in the body is ensured by several mechanisms involving ceruloplasmin and albumin in the liver, as well as copper transport proteins. However, an excess of copper can be toxic to normal cells due to the generation of reactive oxygen species (ROS) or reactive nitrogen species (RNS) [44,45].

Unlike normal cells, tumor cells have reduced vascularity, and therefore, a low oxygen level, which explains the invasion, metastasis and activation of an anaerobic process known as the Warburg effect [46] As a result, tumor hypoxia can be exploited to develop new prodrugs that become active in the reducing environment of cancer cells [47]. In this sense, copper ions become very attractive because they can exist in two different oxidation states in cells. The presence of hypoxia in cancer cells causes the reduction of Cu(II) ions to Cu(I), and thus, allows copper compounds to act as targets at the tumor level. The Cu(I) ion, once formed, catalyzes the production of ROS and RNS, which will ultimately induce pro-apoptotic oxidative stress [48].

In the last two decades, copper coordination compounds have consolidated their position in the design of metallo-drugs, as evidenced by the increase in the number of compounds that have demonstrated their efficacy following in vitro or in vivo testing. Initially, it was assumed that most Cu(II) coordination compounds have a similar mechanism of action to Pt(II) compounds, considering DNA as the main biological target. The newly synthetized complexes were structurally and physico-chemically characterized; then, they were tested via in vitro screening on human cancer cell lines and/or interaction studies with the DNA molecule. Particular attention has been paid to Cu(II) complexes including N,N-diimine ligands (and other extended aromatic planar systems contained in thiosemicarbazone or Schiff base assemblies) due to their binding/intercalating action in the DNA structure. Although damage to the DNA molecule is the primary mechanism by which these copper complexes (and many other conventional chemotherapeutic agents) act, the antiproliferative process does not always guarantee selective cytotoxicity to cancer cells. That is why the studies were directed towards the discovery of compounds with other mechanisms of action, at the level of cellular components [49].

The main mechanisms underlying the antitumor action of Cu(II) complexes are:

- Intercalation—the insertion of a copper complex between two pairs of adjacent bases through van der Waals bonds;
- Interaction with the nucleotides in the chains at the “small cavity” level (“minor groove”) of the DNA molecule;

The oxidative mechanism—the production of ROS or RNS species in the immediate neighborhood of the DNA molecule. Electron spin resonance demonstrated mainly the formation of hydroxyl radicals in the process of reducing the Cu(II) ion to Cu(I) in the presence of reducing agents (e.g., H_2_O_2_). These radicals, along with other ROS, are produced by a Fenton or Haber-Weiss type reaction as follows:

LCu (II) + H_2_O_2_ → LCu (I) + •OOH + H ^+^

LCu (I) + H_2_O_2_ → LCu (II) + •OH + OH

where L, represents an organic ligand.

- In the hydrolytic mechanism, the interactions of Cu(II) ions with the phosphate anion from the nucleotides cause the breaking of the phosphodiester bonds and the destruction of nucleic acid molecules;
- The inhibition of topoisomerase I or II, with a role in DNA replication and transcription;
- Proteasome inhibition (proteasomes represent multiprotein complexes located both in the nucleus and in the cytoplasm, which selectively degrades and recycles intracellular proteins) [50,51,52].

### 2.3. Ruthenium Compounds

The chemical element ruthenium, ^44^Ru, has properties that recommend it as a complex generator in coordinative compounds, with biomedical implications:

- The ability to form labile complexes that can participate in a slow exchange of ligands with molecules in biological environments. Ru(II) and Ru(III) complexes have ligand-exchange kinetics similar to those of Pt(II) complexes. It was demonstrated that the Ru(II) ion has a greater preference for ligands containing S donor atoms, such as glutathione and methionine, and a lower preference for O or N donor ligands [53].
- The ability to have several oxidation states: Ru(II), Ru(III) and Ru(IV); their oxidation or reduction processes are possible under physiological conditions. Ruthenium is unique in the fact that its oxidation states, II, III and IV, are all accessible under physiological conditions. In these oxidation states, the metal ion which generates the complex is hexacoordinated, and the complex geometry is octahedral. Due to this orientation of the ligands, the formation of “intrastand cross-linking” bonds with residues of nitrogenous bases belonging to the same DNA chain encountered in the case of platinum complexes is very low. The bonds that are formed between these Ru complexes and DNA molecules are of the “interstand cross-linking” type, with residues of nitrogenous bases from two different chains. However, this is not the main mechanism of antitumor action. The altered metabolism of cancer cells causes, at their level, hypoxia, a high level of glutathione and a lower pH, which provides the premise for an environment with reducing properties. To increase the selectivity of ruthenium complexes against cancer cells and to reduce, as much as possible, the toxic effects on normal cells, the redox potential of ruthenium can be modified once it enters the body. For example, these agents can be administered as relatively inert Ru(III) complexes (prodrugs), which are then activated by reduction inside the malignant cell [53,54].
- The ability to replace Fe(III) ions transported in the body by various plasma proteins (for example, transferrin).

Due to the structural similarities between Fe(III) and Ru(III) ions, the latter can replace the Fe(III) ion in its site in serum transferrin and albumin (proteins that solubilize and transport iron in plasma). The transport of ruthenium complexes in the blood is an aspect that has attracted the attention of the scientific community since the first studies. The similar chemical behavior of Ru(III) to that of Fe(III) explains the interest in ruthenium compounds for serum proteins, especially for transferrin. This explains the fact that physiological iron transport mechanisms (the so-called “transferrin pathway”) could be exploited by ruthenium species as a smart way to enter cells, according to the “Trojan horse” strategy. Because rapidly dividing cells (including cancer cells) have a greater requirement for iron, there is an increase in the number of transferrin receptors on the cell surface, resulting in more iron-loaded transferrin in the blood circulation. Thus, in vivo studies have shown that there is a 2- to 12-fold increase in the concentration of ruthenium in cancer cells compared to healthy cells. Since ruthenium complexes preferentially target tumor cells, its systemic toxicity is expected to be minimized. Furthermore, it has been shown that ruthenium is transported into cells via both transferrin-dependent pathways and other plasma protein-independent mechanisms [53,55].

In the last 30 years, a large number of ruthenium complexes with octahedral geometry characteristic of Ru(III) and Ru(II) ions, have been synthesized and tested for potential antitumor activity. The initial studies started from the premise that ruthenium will form coordinating compounds that will manifest their antitumor action through direct interaction with the DNA molecule, analogously with platinum compounds. It was later shown that there are significant differences between the two types of antitumor agents in terms of their mechanism of action [17,53].

First, ruthenium complexes seem to accumulate preferentially in neoplastic cells, not in normal ones, using transferrin as a transporter to enter into the tumor cell. The Ru(III) complex remains relatively inactive until it reaches the interior of the target cell where it binds to the transferrin receptor. Here, the reduction of the Ru(III) ion to a Ru(II) ion and an exchange of ligands with molecules from the biological environment take place. The low oxygen content and the more acidic pH in tumor cells favors reduction to the more reactive Ru(II) ion. This process was named “activation by reduction” and results in selective antitumor activity on hypoxic tumors resistant to chemotherapy and radiotherapy. On the other hand, some Ru(III) complexes have shown greater efficiency against metastases than against primary tumors. This antimetastatic effect is explained by inhibition of the detachment of tumor cells, and therefore, the prevention of the formation of a new growth substrate. Given these properties, ruthenium complexes show different patterns of antitumor activity and toxicity to platinum complexes [56,57].

## 3. New Metallo-Drugs in Clinical Trials

The literature abounds with data on new metal complexes with antitumor activity, but few of them have reached preclinical and clinical trials, and very few have passed them. In this review, we stopped only on platinum, copper and ruthenium complexes, presenting only those that have reached clinical trials in different phases, or that have materialized into metallo-drugs [58].

### 3.1. New Platinum Compounds

While cisplatin is used widely in the clinic to treat many tumour types, clinical utility is limited by two major factors: (i) drug resistance, either intrinsic or acquired, and (ii) the principal dose-limiting side effects of nephrotoxicity and neurotoxicity. Unfortunately, although the initial tumour response to cisplatin in many tumours may be high, most patients will nevertheless relapse and die of their disease.

#### 3.1.1. BBR 3464

**Figure 2:**
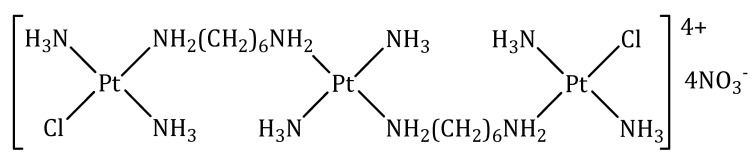
Chemical structure of BBR 3464.

BBR 3464 is a charged (+4), triplatinum complex whose structure derives from that of trans-diammindichloroplatinum(II), in which the bridges between the Pt(II) ions are represented by 1,6-diaminohexane (Figure 2).

BBR 3464 has notable preclinical characteristics. It is approximately fourty to eighty fold more potent than cisplatin on a molar-dose basis and is active in vivo in cisplatin-sensitive and -resistant tumors, as well as in insensitive xenografts. Additionally, BBR 3464 was shown to be more active than cisplatin in *p53* mutant tumors. The characteristic DNA-interaction of BBR 3464 results in the inhibition of DNA replication and RNA transcription, with triggering of the apoptotic cascade leading to cell death. Unlike cisplatin, BBR 3464 leads to prolonged tumor-growth inhibition after the discontinuation of treatment, suggesting that the two drugs could differ significantly in their ability to perturb the cell cycle. As a distinctive feature, BBR 3464 achieves a high proportion of interstrand and intrastrand DNA adducts in contrast to the effects of cisplatin, which predominantly produces the latter type of DNA damage. While cisplatin-damaged DNA is recognized by HMG proteins, the conformational changes resulting from BBR 3464 interaction with DNA are not. This observation might explain the resistance to cisplatin, and the lack of resistance to BBR 3464 in tumors expressing mutations of the *p53* oncosuppressor gene [59].

In vitro and in vivo studies have shown that BBR 3464 manifests its cytotoxic properties at concentrations 10 times lower than cisplatin and is effective on cell lines resistant to it [60].

Preclinical toxicology in mice, rats and dogs treated on a single or five-timed-daily refracted schedule showed that target organs for BBR 3464 toxicity was bone marrow, resulting in leukopenia, while renal tubulopathy was only minimal or slight. A slight pulmonary interstitial reaction, with fibroblast proliferation and inflammatory infiltration, was observed in mice and dogs. This unusual effect was more extensive after intravenous bolus, given as a single or weekly dose, than after slow infusion or a bolus given every two weeks. The schedule dependency of the lung toxicity prompted the clinical evaluation of a five-times-daily refracted schedule in addition to a single dose schedule.

Phase I clinical studies established the value of the maximum tolerated dose as 0.12 mg· m^−2^·day^−1^ at a daily administration rate, for 5 days. The toxicity of this compound is its main disadvantage. In the case of increasing the dose to 0.17 mg·m^−2^·day^−1^, severe myelosuppression and gastrointestinal toxicity were recorded. On the other hand, no nephrotoxic effects were reported [59].

Phase II studies performed on patients with non-small cell lung carcinomas and ovarian tumors in advanced stages highlight the increased efficiency of BBR 3464 compared to therapeutic combinations of platinum compounds with taxanes, but also a lack of activity of the compound against gastric tumors and cancer small cell lung [61,62,63]. In the case of a phase II study regarding the effectiveness of this compound in the treatment of pancreatic cancer, although the completion deadline has been exceeded, the results have not yet been made public [64].

#### 3.1.2. Satraplatin 

**Figure 3:**
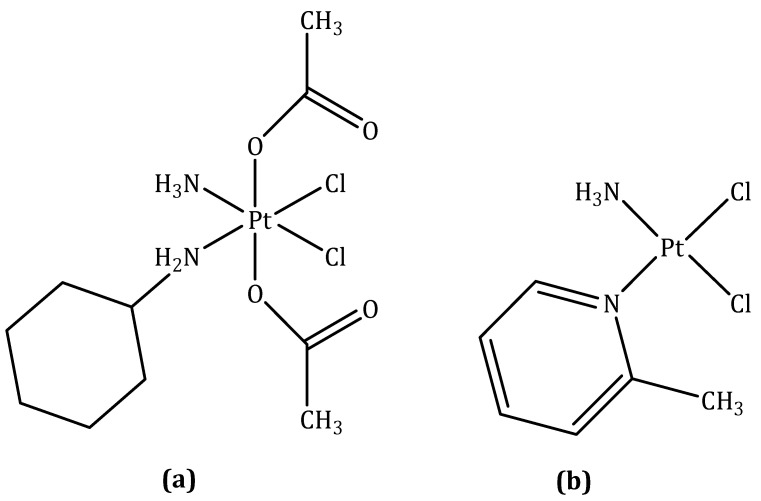
Chemical structure of: (**a**) Satraplatin; (**b**) Picoplatin.

Satraplatin (codenamed JM216), structurally, is [bis-(acetato)-ammine dichloro-(cyclohexyl-amine) platinum(IV) (Figure 3a) and was the first orally active platinum agent to be reported.

Satraplatin was rationally designed such that the lipophilicity and stability were suitable for oral administration. The half-life of reduction of satraplatin by 5 mM ascorbate takes 50 min, which is an adequate time for absorption by the gastrointestinal mucosa in the platinum(IV) form once ingested [21]. Upon entry into the bloodstream, satraplatin undergoes reduction to give six distinct platinum(II) species. Ammine dichloro-(cyclohexyl-amine)platinum(II), derived from the loss of two acetate ligands, is the major metabolite and also exhibits the most potent anticancer activity [65].

Like cisplatin, satraplatin acts through the formation DNA cross-links, DNA distortion, and subsequent inhibition of DNA transcription and replication. The ability of satraplatin to overcome cisplatin resistance is thought to arise from the asymmetric nature of the DNA lesions, which unlike cisplatin adducts, can evade recognition by DNA repair proteins [21]. 

In preclinical studies, satraplatin exhibited a better toxicity profile than cisplatin, and showed activity in cisplatin-resistant human tumor cell lines [66].

In vivo studies in mice bearing murine ADJ/PC6 plasmacytoma, which we note was the same model used to identify carboplatin as a viable alternative to cisplatin, showed satraplatin to exhibit markedly superior antitumor efficacy relative to cisplatin and carboplatin. Also, in four ovarian carcinoma xenograft models of varying cisplatin and carboplatin resistance, satraplatin displayed activity similar to that of cisplatin and carboplatin, which were administered intravenously. In rodents, the dose-limiting toxicity of satraplatin was myelosuppression. 

We emphasize that less hepatotoxicity and fewer gastrointestinal effects were observed as compared to treatment with cisplatin or carboplatin [21]. 

In the first Phase I study, satraplatin was administered at doses ranging from 60–170 mg·m^−2^ as a single oral dose. The pharmacokinetics data suggested that gastrointestinal absorption was being saturated, preventing the maximum tolerated dose from being reached. To improve absorption into the bloodstream, patients were treated on a five-times daily schedule with lower doses (30–140 mg·m^−2^) [67]. The dose-limiting toxicities were thrombocytopenia and neutropenia and in about 10% of the patients treated, nausea, vomiting, and diarrhea were also observed. Based on the Phase I studies, doses of 100–120 and 45–50 mg·m^−2^ were recommended for repeated daily dosing for 5 and 14 days, respectively, in Phase II/III trials [21].

Several Phase II/III trials have been carried out to determine the efficacy of satraplatin. A Phase II study on metastatic NSCLC patients, in which satraplatin was administered as single daily 120 mg·m^−2^ doses for 5 days on 3 week cycles, failed to provide any objective responses. Nevertheless, 46% of the patients were noted to express some palliation. A more advanced Phase II study on patients with small-cell lung cancer and squamous cell head and neck cancer, with escalated doses of satraplatin, produced a response rate of 38%, similar to that observed with cisplatin. Furthermore, this study found no signs of severe neurotoxicity or nephrotoxicity. Other Phase II studies in patients with relapsed ovarian cancer and advanced/recurrent squamous cancer of the cervix produced clinically beneficial or partial rates of response in several patients. The former study noted that the most common form of toxicity were neutropenia and thrombocytopenia [67]. Satraplatin has also been heavily studied as a potential second-line chemotherapeutic for patients with metastatic castration-resistant prostate cancer. Treatment with 120 mg·m^−2^ satraplatin daily for 5 days, used in patients with castration-resistent prostate cancer who had undergone front-line hormone therapy, resulted in 62% of patients expressing stable disease or partial response [68,69]. Currently, a phase I clinical trial is underway regarding the efficacy of satraplatin in the treatment of prostate cancer without metastases [64].

#### 3.1.3. Picoplatin

Picoplatin (codenamed AMD473 or ZD0473), structurally, is diammine dichloro-(2-methylpiridine) platinum(II) (Figure 3b). It was primarily designed to overcome one of the known mechanisms of platinum resistance-detoxification by intracellular thiols—through the introduction of a bulky methylpyridine ring to provide steric hindrance to direct interaction with platinum [58,70]. 

Preclinical evaluation of picoplatin confirmed that this drug was also able to overcome platinum complex resistance in cell lines with high levels of glutathione (GSH) [71]. In studies with human ovarian cell lines, it has been shown that increasing levels of reduced GSH are associated with increasing platinum resistance. In addition, lower levels of glutathione S-transferase (GST) activity have been shown to be associated with enhanced clinical response to platinum-based chemotherapy in head and neck cancer [72]. Additionally, picoplatin was able to overcome resistance because of decreased cellular uptake of the drug in some cell lines or enhanced DNA repair/increased tolerance of platinum adducts in others. Picoplatin forms interstrand cross-links, but does so at a rate intermediate to that of cisplatin and carboplatin, because of its reduced reactivity relative to cisplatin. Using a Taq polymerase stop assay to identify the site of DNA adducts, a novel pattern of DNA binding was identified in pBR322 DNA after incubation with 10 and 100 mM picoplatin for 2 h [71]. This finding may also, in part, account for the observed capability of picoplatin to circumvent adduct tolerance and DNA-repair mechanisms associated with resistance to cisplatin. 

Preclinical pharmacology studies in mice have demonstrated that the maximum tolerated dose is 45 mg·kg^−1^, given as a single intraperitoneal administration, with the dose-limiting toxicity being myelosuppression. Owing to the limited aqueous solubility of picoplatin, preclinical toxicology studies were conducted by intraperitoneal injection following a pharmacokinetic demonstration of the equivalent bioavailability of intraperitoneal and intravenous administration. No renal toxicity was observed. Antitumour activity was noted in several tumour models including human ovarian carcinoma xenografts with acquired resistance to cisplatin and carboplatin. Picoplatin showed an improved antitumour effect compared with cisplatin and satraplatin against the CH1cisR xenograft (34 days growth delay vs 10.4 and 3.5 days, respectively). The antitumour activity was similar when picoplatin was given daily at 60 mg·kg^−1^ for 5 days every week for 4 weeks or by weekly administration (300 mg·kg^−1^ every 7 days for 4 weeks) [73]. 

Results from preclinical studies have highlighted the effectiveness of picoplatin against some types of ovarian cancer, mesothelioma, small cell lung cancer and non-small cell lung carcinomas resistant to cisplatin and oxaliplatin. It is interesting to mention that this compound manages to avoid all three major methods of resistance to cisplatin (deficient absorption, inactivation by thiol compounds and DNA repair mechanisms) [70].

On the basis of the preclinical antitumour activity seen with picoplatin, especially in models with acquired platinum resistance, and its lack of nonhematological toxicity, the drug was taken into clinical development. 

A Phase I trial was carry out at the Royal Marsden Hospital under the auspices of the Cancer Research UK Phase I/II Committee. The initial schedule chosen was a short intravenous infusion given once every 3 weeks. A pharmacokinetically guided dose-escalation scheme was used, for several reasons. Firstly, there is no evidence of metabolism of picoplatin in vivo, and thus, the free platinum AUC (area under the concentration vs. time curve) should reflect the biologically important species in both man and mouse. Secondly, previous experience with cisplatin and carboplatin has demonstrated the close relation between the AUC at maximum tolerated dose in mice and humans. It was hoped that this approach would reduce the number of dose escalations required to reach the maximum tolerated dose [74].

Following phase I studies, the maximum tolerated dose value was established as 150 mg·m^−2^ and the picoplatin administration regimen as doses of 120 mg·m^−2^, intravenously, once every 3 weeks, with the possibility of increasing the dose for patients who had not previously undergone antitumor treatment. The limiting toxic effects were neutropenia and thrombocytopenia, whereas alopecia, neurotoxicity and nephrotoxicity were not reported. Other adverse effects included nausea and vomiting, anorexia and metallic taste [58].

Phase II clinical trials in patients suffering from various types of lung tumors resulted in a response rate of 15.4% in small cell lung cancer resistant to other platinum compounds [75]. Other phase II studies have highlighted the fact that picoplatin is also active on ovarian tumors sensitive and resistant to other platinum compounds, bringing a benefit in terms of survival rate and the limitation of disease progression [76]. Similar results have been reported in the case of patients with mesothelioma and metastatic breast cancer; for approximately 50% of patients, a stop was found in the progression of the disease [77,78]. In the case of some phase II studies regarding the effectiveness of picoplatin in the treatment of colorectal and prostate cancer refractory to hormone therapy, although the completion deadline has been exceeded, the results have not yet been made public [64].

Only one phase III study was undertaken targeting small cell lung cancer. The study showed that patients who did not respond to treatment up to that point or who had experienced rapid disease progression benefited from an extension of life after treatment with picoplatin [32].

#### 3.1.4. Ormaplatin

**Figure 4:**
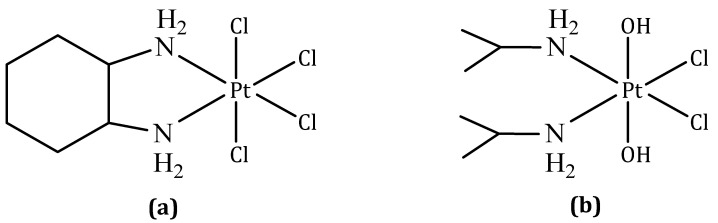
Chemical structure of: (**a**) Ormaplatin; (**b**) Iproplatin.

Ormaplatin (also known as tetraplatin, codenamed NSC 363812), structurally, is tetrachloro (1,2-diaminocyclohexane) platinum(IV) (Figure 4a).

Ormaplatin is rapidly reduced to the corresponding dichloro (1,2-diaminocyclohexane) platinum(II) form in tissue culture medium (t_1/2_ = 5–15 min) and undiluted rat plasma (t_1/2_ = 3 s) [21]. The active platinum(II) species is similar to oxaliplatin. Ormaplatin displayed in vitro and in vivo activity against some cisplatin-resistant cancers and was taken forward to clinical trials [79]. Various doses, dose patterns, and modes of administration (intravenous and intraperitoneal) were investigated in six Phase I clinical trials; however, no Phase II clinical trials have been planned [80,81]. Ormaplatin was found to induce severe neurotoxicity at the maximum tolerated dose, and in some cases, a safe maximum tolerated dose could not be determined. Toxicity is thought to arise from fast reduction to the active platinum(II) form as a consequence of the axial chloride ligands [21].

#### 3.1.5. Iproplatin

Iproplatin (also known as JM9 or CHIP), structurally, is dichloro-dihydroxy-bis(isopropylamine) platinum(IV) (Figure 4b). Iproplatin is structurally similar to ormaplatin in the sense that it contains two equatorial chloride groups which are cis to each other [21]. 

Carbon-14 labelling studies showed that the mechanism of action of iproplatin involves the reduction of the platinum(IV) center to platinum(II) followed by covalent bond formation with DNA. Iproplatin is less prone to reduction and deactivation by biological reducing agents than ormaplatin, presumably because of the presence of hydroxide axial ligands, allowing less hindered distribution throughout the body. Another advantage of iproplatin is its very high water solubility (44.1 mM), which allows simpler formulation and administration [82]. 

Iproplatin is one of the most clinically studied platinum agents to have not been approved for marketing, with 38 clinical trials ranging from Phase I to III having been concluded. Phase I studies revealed that the dose-limiting toxic effect was myelosuppression, which, in one study involving children, was partly correlated with the amount of prior therapy chemo- and radiotherapy received. The same study recommended intravenous doses of 324 mg·m^−2^ over 2 h every 3–4 weeks for Phase II trials in children. Other studies proposed doses of 45–65 mg·m^−2^ and 95 mg·m^−2^ for patients treated on a five-times-daily schedule every three weeks and a four-times-weekly schedule with two-week break periods, respectively [83]. Phase II trials were carried out in patients with a variety of different cancer types, and Phase III trials were conducted in ovarian cancer patients and those with metastatic epidermoid carcinoma of the head and neck [84,85]. The ultimate conclusion of these studies was that iproplatin did not exhibit overall effectiveness that surpassed that of cisplatin or carboplatin and no further trials were undertaken [21].

### 3.2. New Copper Compounds

The differential response of normal and tumor cells exposed to Cu(II) ions is the starting point for obtaining new compounds with antineoplastic properties. Many of the copper complexes are active against tumor cell lines resistant to cisplatin and analogous compounds, and exhibit lower toxicity than established platinum derivatives as antitumor agents. 

Preclinical and clinical studies provide encouraging evidence to support the therapeutic potential of copper complexes despite their high toxicity. Due to the promising results obtained from in vitro and in vivo testing, some of these complexes have reached the clinical testing phase. In this context, Cu(I) and Cu(II) complexes present encouraging perspectives [86].

#### 3.2.1. Elesclomol

**Figure 5:**
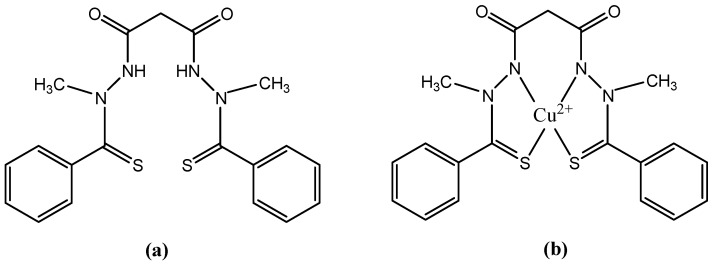
Chemical structure of: (**a**) Elesclomol; (**b**) Complex Cu(II)-Elesclomol.

Elesclomol (codenamed STA-4783), structurally, is N-malonil-bis(N-metil-N-tiobenzoyl hidrazide). The structural formulas of elesclomol and its Cu(II) complex are shown in Figure 5.

Elesclomol is an injectable, small molecule. It has been developed as a sodium salt formulation for single-agent use or for combination use with other anti-cancer drugs. The free acid form of elesclomol is the active ingredient in both elesclomol and sodium elesclomol. While in the bloodstream, elesclomol binds to copper (II) ions present in the serum. Cancer cells efficiently take up this complex, unlike free elesclomol. Once inside the cell, the copper in the complex undergoes a redox reaction whereby Cu(II) is reduced to Cu(I). This reaction, which is mediated by elesclomol, creates ROS and oxidative stress in the cell. The anti-cancer activity of elesclomol is attributed to its ability to directly increase this oxidative stress [87]. Cancer cells already have an elevated level of oxidative stress relative to most normal cells. It was hypothesized that the further increase in ROS induced by elesclomol would exceed a critical threshold in cancer cells, enhancing the sensitivity to traditional cytotoxic chemotherapeutic agents and triggering tumor cell death while sparing most normal cells [88].

Elesclomol exerts its activity by disrupting the metabolism of mitochondria in cancer cells. This activity requires the presence of oxygen that results in energy metabolism being driven primarily through oxidative phosphorylation in mitochondria. Under hypoxic conditions, energy metabolism occurs through glycolysis in cytoplasm, rather than in mitochondria. Under hypoxic conditions, often associated with elevated lactate dehydrogenase (LDH) levels, elesclomol’s activity is diminished. These observations are consistent with findings in a phase 3 metastatic melanoma study, where elesclomol activity was found only in subjects with normal baseline LDH levels [87,88,89]. 

Elesclomol and Cu(II)-elesclomol are both highly active in vitro and typically inhibit tumor cell growth at low (nanomolar) concentrations. In preclinical models, elesclomol has demonstrated synergistic anti-tumor activity with both paclitaxel and docetaxel, as well as single-agent activity [88]. Elesclomol showed antitumor activity against a broad range of cancer cell types and substantially enhanced the efficacy of chemotherapeutic agents such as paclitaxel in human tumor xenograft models [90]. 

In a phase I clinical trial, in combination with paclitaxel in patients with refractory solid tumors, elesclomol was well tolerated, with a toxicity profile similar to that observed with single agent paclitaxel [91]. 

In a double-blinded, randomized, controlled phase II clinical trial in 81 patients with stage IV metastatic melanoma, elesclomol, in combination with paclitaxel doubled median progression-free survival compared with paclitaxel alone. Cutaneous melanoma is a highly malignant tumor derived from melanocytes, the pigment-producing cells in the epidermis of the skin. If diagnosed and surgically removed while localized in the outermost skin layer, melanoma is potentially curable. However, for patients with deeper lesions or metastatic disease, the prognosis is poor, with an expected median survival of only 6 to 9 months for patients with stage IV metastatic melanoma [92]. Interestingly, under hypoxic conditions, often associated with elevated LDH levels, the activity of elesclomol is diminished. Thus, the combination of elesclomol with paclitaxel proved effective only in patients with normal LDH levels, and no changes were observed in those with elevated LDH levels. Despite intensive efforts to improve disease prognosis, little progress has been made [87,90].

In another phase I study, elesclomol, given in combination with paclitaxel in women with refractory ovarian cancer, obtained a favorable opinion at this stage, with the combination being well tolerated. Currently, elesclomol combined with paclitaxel is in a phase II study in patients with ovarian, fallopian tube or peritoneal cancer that is recurrent or resistant to cisplatin treatment. Only patients with normal LDH levels were selected for the study [88]. Researchers have also planned to start a phase I study for the combined administration of elesclomol+docetaxel+prednisone in patients with metastatic prostate cancer [93].

#### 3.2.2. Casiopeinas: Casiopeina III and Casiopeina II-gly

**Figure 6:**
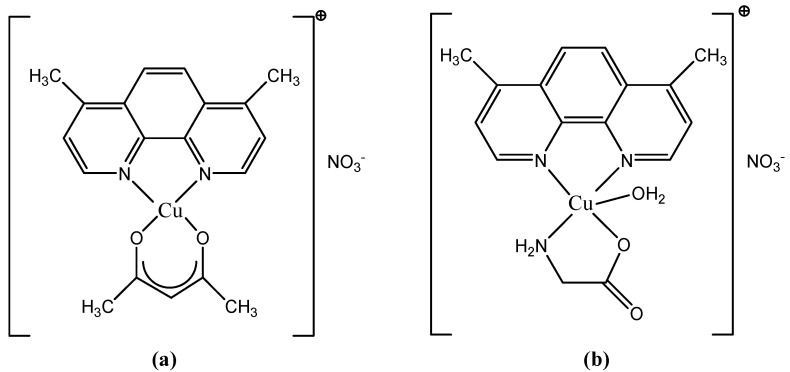
Chemical structure of Casiopeinas: (**a**) Casiopeina III; (**b**) Casiopeina II-gly.

Structurally, Casiopeinas are mixed Cu complexes with the general formula [Cu(N-N)(X-Y)H_2_O]NO_3_, where N-N is a diimine ligand (phenanthroline or dipyridyl) and X-Y is a bidentate ligand (acetylacetone, salicylaldehyde, peptide, benzimidazole). The representatives selected for preclinical and clinical testing are Casiopeina III (CasIII) and Casiopeina II-gly (CasII-gly). Their structural formulas are presented in Figure 6.

These are chelated complexes of Cu(II) with 4,7-dimethyl-1,10-phenanthroline and glycocol (CasII-gly) or with 4,7-dimethyl-1,10-phenanthroline and acetylacetone (CasIII) [55].

Casiopeinas are a group of copper-based chemical compounds with cytotoxic, genotoxic, antiproliferative and antineoplastic activity, as demonstrated in vitro and in vivo [42]. Casiopeinas were developed based on the rationale that copper compounds, unlike other metallic-based therapies, are more readily metabolized; this property decreases the incidence of side effects found in several other chemotherapies [94]. 

The main cytotoxic effect that representatives of this class have demonstrated is the activation of pro-apoptotic processes in malignant cells. There are at least two ways in which these compounds act on tumor cells: intercalation into the DNA structure followed by preventing its proper replication, and biochemical mechanisms leading to programmed cell death (apoptosis). Casiopeinas increase the level of ROS produced near DNA, most likely through a redox mechanism involving the Cu(II) ion. These species, once produced, will react with the DNA molecule causing damage to its structure most often by launching a radical attack at the level of the deoxyribose residue. Additionally, by increasing the level of ROS in the mitochondria, dysfunctions occur through the oxidation of thiol residues at the level of mitochondrial proteins and, finally, cell apoptosis occurs. Other mechanisms of action attributed to this class of Cu(II) chelate complexes, discovered later, involve DNA fragmentation and cell death through caspase-dependent pathways, or inhibition of energy metabolism and mitochondrial toxicity. In addition, CasII-gly exhibits an anti-tumor effect by inhibiting the cell cycle, regulating the transformation processes in fibroblasts or reducing the phenomenon of uncontrolled migration of tumor cells [94,95].

CasIII was entered into a phase I clinical trial, to test for acute myeloid leukemia and colon cancer. CasII-gly, because it blocks the migration and proliferation of HeLa cells, was entered into clinical trials to treat cervical cancer [96].

CasII-gly is currently in phase I clinical trials looking at its toxicity in humans. Tests have shown that it inhibits energy metabolism and induces a high cardiotoxic effect, a fact that will probably cause the clinical trials to be stopped [42,55]. 

The advantage observed in the case of clinical trials performed on Casiopeinas consists in an increase in the immunity of the patients; this is accompanied by a mechanism of high protection of the liver through the repair of damaged cells, compared to other cytotoxic drugs. A CasII-gly-based therapy may prevent the problematic side effects of chemotherapy, which often compromise patients’ health [54].

### 3.3. New Ruthenium Compounds

The success of cisplatin stimulated the scientific world in the development of other metal complexes with antitumor activity that are superior to platinum complexes, have lower toxicity or are active on other types of tumors. In this context, ruthenium complexes seem to be promising. Two such compounds have been entered into clinical trials. Despite their structural and chemical similarities, the two Ru(III) complexes show distinct antitumor behaviors. In preclinical studies, NAMI-A has demonstrated an inhibitory effect against the formation of cancer metastases in a variety of animal tumor models, but does not show direct cytotoxic effects on primary tumors, while KP1019 exhibits antitumor activity against a wide range of primary tumors humans through a cytotoxic mechanism of apoptosis induction [57,97,98,99].

#### 3.3.1. NAMI-A

**Figure 7:**
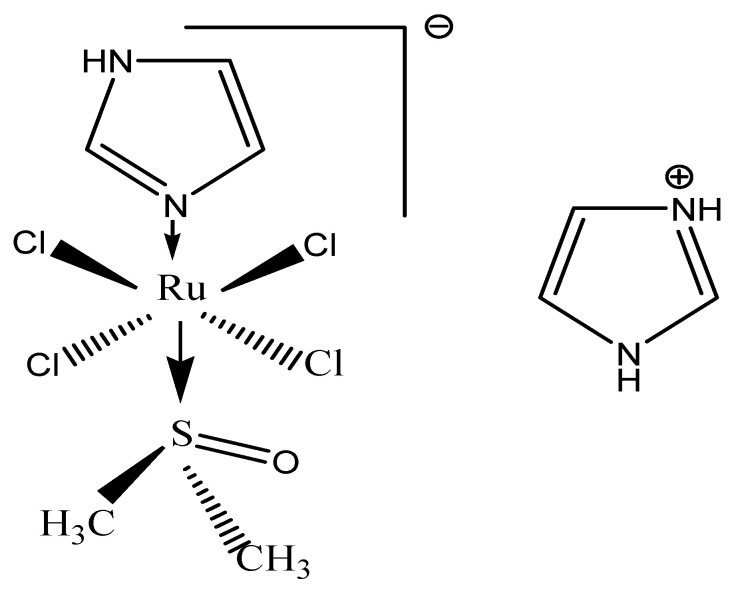
Chemical structure of NAMI-A.

Structurally, NAMI-A is [ImH][trans-RuCl_4_(DMSO)(Im)] (Im=imidazol, DMSO=dimetilsulfoxid) (Figure 7).

The proposed mechanisms of action of NAMI-A in metastasis control include the following: limiting actin dependent adhesion in vitro [100]; limiting in vitro tumor cell motility via cytoskeleton remodeling (the activation of collagen receptor integrin β1 on the cell surface results in RhoA activation and, subsequently, in rearrangement of the cytoskeleton in vitro [101,102]; and exerting anti-invasive effects in vitro and in vivo by promoting capsule formation (NAMI-A increases the extracellular matrix around tumor cells and tumor vasculature by triggering fibrotic reactions, regulates TGFβ1 expression, binds to collagen and stimulates collagen production [103,104,105] and anti-angiogenic effect (e.g., NAMI-A inhibits the angiogenic effect induced by vascular endothelial growth factor in vitro) [106].

NAMI-A transiently blocks cell cycle progression in vitro at G2M phase [107,108]. The mechanism might be activation of Chk1, resulting in the inhibition of CDC25 and, subsequently, in inactive phosphorylated CDC2, thereby preventing mitotic entry [101].

In vitro, NAMI-A inhibits the mitogen-activated protein kinase/extracellular signal-regulated kinase (MAPK/ERK) signaling pathway and c-myc transcription [109,110]; DNA binding—although the intrastrand adduct formation of NAMI-A is significantly less than that of cisplatin, Ru-G and Ru-AG intrastrand adducts—was observed in vitro [111]. The AG:CG adduct ratio was four times higher for NAMI-A compared to cisplatin. NAMI-A sporadically forms interstrand crosslinks, whereas the formation of DNA protein crosslinks is comparable to cisplatin [101]. 

Due to its fast ligand exchange kinetics, it was found that NAMI-A is not significantly internalized by cells, but rather, binds to extracellular collagen matrix and to cell surface integrins, leading to increased adhesion and reduced cancer cell spread. If these results are consistent with the ability of NAMI-A to inhibit the growth of new metastases, its activity against already-developed metastases is probably due to its antiangiogenic properties [112].

Contrary to cisplatin, the cytotoxic effect of NAMI-A is not remarkable (on average, 1053 times less than cisplatin) [113]; its cytotoxicity has been found to be correlated with DNA binding (which is also the case for cisplatin) [101,111]. 

In preclinical studies, administration of NAMI-A in more frequent smaller dosages showed more prominent antimetastatic effects. Notably, the action of NAMI-A seems to be independent of the type of primary tumor or the stage of growth of metastases. NAMI-A is capable not only of preventing the formation of metastases, but also of inhibiting their growth once established [56]. Preclinical animal studies using NAMI-A have shown selective activity against lung metastases from a variety of primary tumors in murine models. NAMI-A reduced the weight of lung metastases more than their number. Since larger concentrations of NAMI-A in the lungs than in other tissues was ruled out, this finding was assumed to be related to the selective interference of NAMI-A with the growth of metastases already established in the lungs [114].

Toxicologic studies in dogs and mice have revealed an acceptable toxicity profile. The calculated half-life was approximately 18 h. Toxicity was observed at concentrations greater than 50 mg/kg/day, and in mice that survived treatment, was reversed within 3 weeks of the end of the treatment [115]. 

NAMI-A was the first ruthenium compound which entered into clinical trials. A phase I study was performed with NAMI-A as a single agent, given as an infusion over 3 h daily for 5 consecutive days every 3 weeks in patients with different types of solid tumors (including colorectal cancer, non-small cell lung cancer, melanoma, ovarian cancer, pancreatic cancer and mesothelioma). In total, 24 patients were treated at 12 different dose levels (2.4–500 mg/m^2^/day). All 24 patients underwent refractory to conventional treatment. The advised dose for further testing was 300 mg/m^2^/day. Adverse events included only mild hematologic toxicity, quite disabling nausea, vomiting, and diarrhea; furthermore, patientsexperienced stomatitis, fatigue, common toxicity criteria grade 1 and 2 creatinine increase, fever and sensitivity reactions to NAMI-A. Finally, phlebitis at the infusion site was observed when NAMI-A was administered intravenously without a port-a-cath. There was also painful blister formation on hands, fingers and toes, although no part of the formal common toxicity criteria was considered dose-limiting. Twenty out of twenty-four patients were evaluable for response evaluation. One patient (4%) with non-small cell lung cancer experienced stable disease for 21 weeks, and nineteen patients (79%) showed disease progression. The transport of NAMI-A was achieved via binding to plasma proteins. It was found that NAMI-A accumulates in white blood cells, but accumulation was not directly proportional to daily dose or total drug exposure. Studies of this phase have shown stabilization of the disease in patients with advanced lung cancer. This result, together with the excellent activity shown by NAMI-A against lung metastases in mice, led to the recommendation of NAMI-A for a phase II study in the treatment of non-small cell lung cancer. As the combination of cisplatin and gemcitabine is widely used for the first-line treatment of non-small cell lung cancer, researhers decided to test a similar combination with NAMI-A [56,107,112].

A phase I/II study in which NAMI-A was given in combination with gemcitabine to 32 patients with advanced non-small cell lung cancer was performed. Phase I of the study was directed towards establishing the optimal dose of the combination of NAMI-A and gemcitabine (given at the typical dose of 1000 mg m^–2^). The maximum tolerated dose of NAMI-A was found to be 300 mg· m^–2^ in the 28-day cycle (3 h infusion on days 1, 8, and 15, and gemcitabine given on days 2, 9, and 16) and 450 mg·m^–2^ in the 21-day cycle (NAMI-A administered on days 1 and 8, and gemcitabine on days 2 and 9). A further dose escalation of NAMI-A to 600 mg m^–2^ was found to induce dose-limiting toxicity. Besides neutropenia, the main non-hematological adverse events involved elevated liver enzymes, transient creatinine elevation, nausea, vomiting, constipation, diarrhea, fatigue and renal toxicity. Blister formation on fingers was observed only at 600 mg· m^–2^. 

The 21-day regimen was used for the phase II part of the study, in which 15 patients were treated with the maximum tolerated dose of NAMI-A established in phase I with the aim of assessing the antitumor activity according to the response of evaluation criteria for solid tumors [116]. Out of the 27 patients evaluable for response, partial remission was observed in one case (at 300 mg·m^–2^ in the 21-day schedule) and stable disease for at least 6–8 weeks in 10 patients. These results were not sufficient to warrant further expansion of the phase II cohort with additional 12 patients. Overall, the efficacy of the treatment was lower than expected for gemcitabine alone and it was declared to be “insufficiently effective for further use” [107,112].

#### 3.3.2. KP1019 and KP1339 

**Figure 8:**
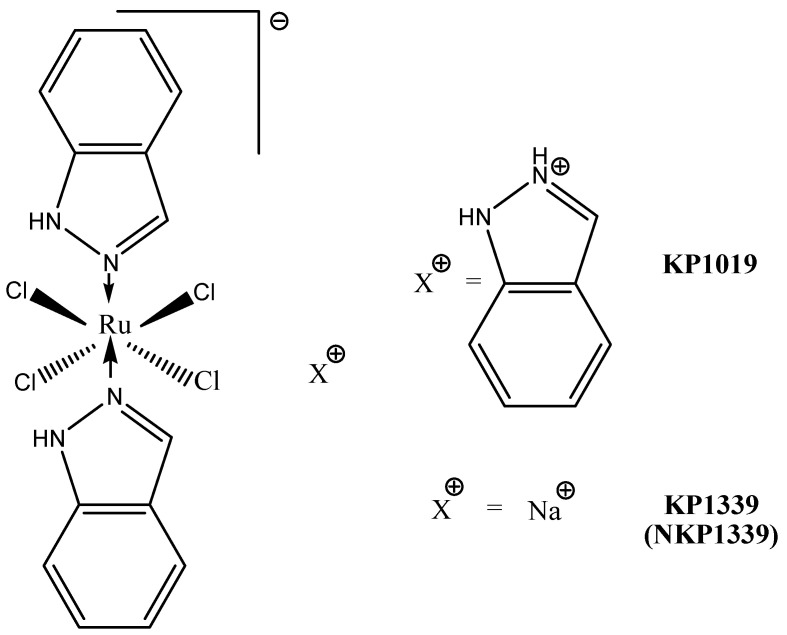
Chemical structure of KP1019 and of it’s sodium salt, KP1339.

Structurally, KP1019 is [InH][trans-RuCl_4_(In)_2_] (In = indazol) and KP1339 it is KP1019 sodium salt (Figure 8).

The mode of action of KP1019 is very distinct from that of NAMI-A. These differences probably arise from the observed kinetic differences in the aquation processes and ruthenium activation. A key point is represented by the large difference in ruthenium uptake in the two cases, leading to significantly higher ruthenium concentrations in the cytosol for KP1019. Accordingly, the in vivo activity of KP1019 on primary tumor growth is believed to be predominantly due to cytotoxic effects on tumor cells arising from direct interference with cell signaling and metabolic pathways; in other words, KP1019 behaves as a classical cytotoxic drug. Furtheremore, one of the recent interpretation of the molecular mechanism of KP1339 tends to rule out direct DNA damage as the main determinant of its cytotoxic action. In contrast, the postulated mode of action involves strong interactions with cytosol proteins, leading to ROS overproduction, oxidative stress and endoplasmic reticulum stress through targeting the chaperone protein GRP78. Eventually, this cellular damage triggers apoptosis through a mitochondrial pathway [117,118].

Based on a quite complex investigative strategy relying on transcriptomics and a genetic screening approach in a budding-yeast model, various genetic targets and a plethora of cellular pathways targeted by KP1019 were identified. Then, the actions produced by KP1019 in yeast were compared with those produced by the same ruthenium drug in Hela cells, and reasonable extrapolations were made. On the grounds of the obtained results, a comprehensive model depicting the mode of action of KP1019 and the targeted cellular pathways was proposed. According to this model, KP1019 induces ROS generation, causes DNA damage (and thus cell cycle arrest), activates mitogen-activated protein kinase signaling, alters intracellular metal ion and lipid homeostasis and also affects the chromatin assembly. Cells activate transcriptional responses to alleviate cellular damage [119]

Moreover, it was found that the toxicity potential of KP1019 is enhanced in the presence of various metal ions but suppressed by the supplementation of Fe^2+^ ions, and reduced glutathione, *N*-acetylcysteine, and ethanolamine. Thus, this model postulates for KP1019 a realistic multifactorial mechanism mediated by a variety of yet-unknown molecular targets [99]. 

KP1019 is moderately cytotoxic in vitro. Therefore, when tested against a panel of chemosensitive cell lines and their chemoresistant sublines, IC50 values in the range of 50 to 180 µM were measured. When compared with its sodium salt, KP1339, in a few cancer cell lines, KP1019 tended to be moderately more cytotoxic. Nevertheless, the significant correlation between the cytotoxicity profiles suggests that the two complexes have similar modes of action. For both compounds, no correlation between total ruthenium uptake and cytotoxicity was found [120]. Both KP1019 and KP1339 were found to be moderately cytotoxic (30–95 µM), but more cytotoxic than cisplatin, in SW480 and HT29 colorectal carcinoma cells, upon 24 h exposure; moreover, they induced apoptosis, predominantly via the intrinsic mitochondrial pathway. Upon 72 h exposure, cisplatin is much more efective than the two ruthenium compounds [121]. Studies concerning the antimetastatic ability of KP1019 in vitro gave controversial results. The complex revealed some anti-invasive activity in monolayer cultures of breast cancer cell lines, causing the significant reduction of cell migration and invasion [122].

KP1019 was tested in vitro against more than 50 primary tumors explanted from humans; in this highly predictive model, the complex proved a positive response rate higher than 70% [99].

KP1019 has antitumor activity in colon cancer in rats. Treatment with KP1019 resulted in a 95% reduction in tumor volume, with no mortality and no significant weight loss. In addition, its efficacy was superior to 5-fluorouracil, the standard agent used against colorectal cancer [123].

The time-dependent tissue distribution of KP1339 (given i.v.) in non-tumor-bearing BALB/c nude mice was recently determined [124]. The highest (and comparable) ruthenium concentrations were found in the liver, lungs, kidneys and thymus, followed by the spleen and colon (~50% less). Consistent with the trend of total ruthenium in blood plasma, the peak levels in the mentioned tissues were found one to six hours after administration and decreased slowly with time, with the exception of the spleen, where the highest amount was found 24 h post-injection. Based on this promising activity, the two ruthenim compounds were selected for further clinical evaluation [99].

A preliminary phase I dose-escalation study was performed with KP1019 (total doses from 25 to 600 mg) on only eight patients with advanced solid tumors [125]. KP1019, given i.v. twice a week over 3 weeks, was well tolerated in the investigated dose range, and only mild toxicity was observed [126]. Disease stabilization for 8 to 10 weeks, unrelated to the dose, was observed for five out of six evaluable patients. The maximum tolerated dose of KP1019 could not be determined due to insuffcient solubility (too large a volume of infusion solution required for further dose escalation). Therefore, a full-scale phase I study was later performed on 34 patients with the more soluble sodium derivative KP1339. The investigation comprised nine dose levels (20–780 mg/m^2^/day), and KP1339 was given by i.v. infusion on days 1, 8, and 15 in a 28-day cycle [123]. Only minor adverse effects were observed. Grade 2–3 nausea, together with increased creatinine levels, was found to be dose-limiting toxicity at the highest dose. Stable disease up to 88 weeks was found for seven patients with different types of tumors, including two cases of non-small cell lung cancer, and one patient with a neuroendocrine tumor had a partial response. Then, the phase I clinical investigation was repeated on 46 patients, with the same dose levels and treatment schedule [127]. The maximum dose tolerated was established to be 625 mg/m^2^. Addtionally, the tolerability and safety profile were similar to those prior established; no significant hematological toxicity or neurotoxicity were found, with the main adverse events being clinically manageable grade ≤2 nausea, fatigue, and vomiting. KP1339 showed moderate antitumor activity, with a 26% disease control rate targeting three of five patients with carcinoid neuroendocrine tumors and a partial response in one patient with colon cancer [99].

In conclusion, NAMI-A and KP1019/KP1339 are suited for pharmacological investigations, although their stability is not high, and they undergo facile chemical transformations. Their behavior is typical of classical prodrugs, and similar to that of cisplatin and related platinum drugs. In addition, these compounds manifest an acceptable solubility in biological fluids, and their toxicity is limited and tolerable.

The fact that both compounds have been investigated in clinical trials producing scarce evidence of systemic toxicity increases their chances of clinical use. Even though their anticancer effects seem to be rather limited when they are used as standalone agents, there is still the chance to explore a larger number of cancer models, and also use these compounds in combination therapies [99].

## 4. Future Perspectives

Although platinum drugs have brought major advances in oncology, their clinical success is often hindered by the adverse side effects and development of resistance. Additional obstacles include low bioavailability and low water solubility. The strategies for the development of new platinum compounds, which take advantage of the affinity of the Pt(II) ion towards DNA while substantially modifying the mode of action, are multiple and complex. They include:

- The design of Pt(II) compounds target tumor cells by including in the coordination sphere some structures that promote a specific interaction with receptors on the surface of the tumor cell (receptors for glucose, estrogens, etc.). An example is C6-glucose-Pt(II) type conjugates, which could be efficiently transferred intracellularly by glucose transporters, promoting, in this way, preferential accumulation in tumor cells [21,128];
- Compounds that do not bind covalently to DNA, but contain a ligand capable of intercalating between pairs of adjacent nitrogenous bases (ligands with extended aromatic structures: 1,10-phenanthroline, bipyridine, quinoxaline derivatives, etc.) [21,61];
- Compounds of Pt(IV) which, following the intracellular reduction process, release, on the one hand, active species of Pt(II), and on the other hand, ligands with biological activity (dual-threat prodrugs)—for example, complexes with ethacrynic acid (inhibitor of glutathione-S-transferase, the enzyme that catalyzes the binding of platinum compounds to glutathione and their inactivation) and with valproic acid (inhibitor of histone deacetylase, which causes cell differentiation and apoptosis) [21,24,128];

Recently, Pt(IV)-based anticancer prodrugs have been studied extensively, with the aim of reducing the side effects and drug resistance associated with the original Pt(II) anticancer drugs such as oxaliplatin [22,23,24,25,26,27,28]. The administration of Pt(IV) prodrugs, however, still faces significant challenges. For example, small-molecule Pt(IV) prodrugs may not be stable enough in the circulation system and are also limited by an insufficient circulatory half-life, which reduces Pt accumulation in tumor tissues, and thus, restricts the drug efficacy [29,30]. Additionally, conventional Pt(IV) prodrugs do not have the property of controllable activation, leading to adverse effects in off-target tissues [129,130,131].

- Non-toxic compounds that are activated at the target by enzymes that are found in much higher concentrations around tumor tissues—for example, complexes of platinum with cephalosporins, activated by β-lactamases, and complexes with β-glucuronyl, activated by β-glucuronidase [132];
- Compounds for photodynamic therapy (non-toxic, stable in the presence of thiol compounds); they are activated following light irradiation at a certain wavelength, generating active species of Pt(II) and ROS [24,128].

Photoactivatable Pt(IV) antitumor agents represent a promising area for new drug development. Small-molecule photoactivatable Pt(IV) prodrugs have been developed; their activation wavelengths still fall within the visible spectrum and are subject to limited penetration depths. Furthermore, although platinum drugs such as oxaliplatin have been reported to induce immunogenic cell death (ICD), most of these studies were carried out in vitro. The prolonged circulation of oxaliplatin may significantly enhance the associated immune response induced in vivo. Taken together, a comprehensive study to obtain an NIR-activatable and clinical drug-based Pt(IV) complex that exhibits enhanced circulation and elicits an elevated immune response in vivo is warranted. The development of photoactivatable prodrugs of platinum-based antitumor agents is aimed at increasing the selectivity, and hence, lowering toxicity of this important class of antitumor drugs. These drugs could find use in treating localized tumors accessible to laser-based fiber-optic devices. Pt(IV) complexes appeared attractive because these octahedral complexes are usually substitution-inert and require reduction to the Pt(II) species to become cytotoxic. Based on the knowledge of Pt(IV) photochemistry, Pt(IV) analogs of cisplatin, [Pt(en)Cl_2_] and transplatin were designed, synthesized and investigated for their ability to be photoreduced to cytotoxic Pt(II) species. Two classes of photoactivatable Pt complexes have been looked at thus far: diiodo-Pt(IV) and diazido-Pt(IV) diammine complexes. The first generation, diiodo-Pt(IV) complexes, represented by [Pt(en)(I)_2_(OAc)_2_], react to visible light by binding irreversibly to DNA and forming adducts with 5′-GMP in the same manner as [Pt(en)Cl_2_]. Furthermore, the photolysis products are cytotoxic to human cancer cells in vitro. However, these complexes are too reactive towards biological thiols (i.e., glutathione), which rapidly reduced them to cytotoxic Pt(II) species, thus making them unsuitable as drugs. The second generation, diazido-Pt(IV) complexes, represented by cis, trans, cis-[Pt(N_3_)_2_(OH)_2_(NH_3_)_2_] and cis, trans-[Pt(en)(N_3_)_2_(OH)_2_], are also photosensitive, binding irreversibly to DNA and forming similar products with DNA and 5′-GMP in the presence of light as the respective Pt(II) complexes. Recently the Pt(IV) prodrug trans, trans, trans-[Pt(pyridine)2(N3)2(OH)2] (Pt1) and its coumarin derivative trans, trans, trans-[Pt(pyridine)2(N3)2(OH)(coumarin-3-carboxylate)] (Pt2) were discovered as agents for photoactivated chemotherapy. These complexes are inert in the dark but release Pt(II) species and radicals upon visible light irradiation, resulting in photocytotoxicity toward cancer cells [129,130,131].

Some authors suggest that all these approaches basically have the same mechanism of action, namely that of cisplatin, and therefore their success is debatable. More recently, a different mechanism of action has been demonstrated for nanocrystals or nanoclusters based on platinum ions, and hence, these structures seem promising for the success of targeted and selective therapies or in combating the resistance phenomena associated with chemotherapeutics based on platinum ions [22].

Although there have been many studies performed on Cu(II) ion complexes with antitumor activity, a relatively small number of compounds entered the phase of in vitro and in vivo studies, mainly due to their low solubility and bioavailability. Solving the solubility problems of the complexes that end up being tested, from the point of view of biological activity, can be tried by different methods: obtaining inclusion complexes between the cyclodextrin and the metal complex, or the synthesis and characterization of nanoparticles as delivery systems for Cu (II) complexes [133]. Moreover, the corroboration of empirical screening methods with new knowledge from genome and proteome research could allow the progress, from the simple synthesis of cytotoxic agents, with unknown mechanisms of action, to the rational design of active principles based on metal complexes [42,95,96].

Possible cytotoxicity mechanisms, such as DNA damage, DNA intercalation, telomerase inhibition and apoptosis induction, have been investigated on Cu(II) and Cu(I) complexes with 2-thioxoimidazolones as ligands. ROS formation in MCF-7 cells and three-dimensional (3D) spheroids was proven using the Pt-nanoelectrode. Drug accumulation and ROS formation at 40–60 μm spheroid depths were found to be the key factors for drug efficacy in the 3D tumor model, governed by the Cu(II)/Cu(I) redox potential [134].

Ruthenium compounds have been extensively studied over the past two decades from both chemical and biological perspectives.

The unique characteristics of Ru(II) or Ru(III) ions make their compounds attractive antitumor agents through: their selectivity for accumulation in tumor cells through interaction with transferrin receptors; activation via reduction, from inactive Ru(III) to active Ru(II); favorable ligand exchange kinetics; antimetastatic effects through inhibiting tumor cell detachment and migration; and different binding modes to the DNA molecule compared to platinum complexes (due to different geometries). These distinct behaviors have led researchers to recommend ruthenium complexes as potential antitumor agents, useful in malignancies resistant to treatment with platinum derivatives, as they have a different mechanism of action and low toxicity. Optimizing the physico-chemical parameters of the complexes by improving their stability and their ability to directly target cancer cells, ruthenium complexes may constitute an alternative to consider for antitumor therapy [57,97,107].

Transport and target delivery systems with metallo drugs as antitumor agents:
  However, although effective, these drugs have a number of shortcomings: limited spectrum of activity, high toxicity, significant side effects, resistance of some types of tumors, low solubility in water, low bioavailability and short retention time in the blood circulation. Research on obtaining new antitumor metallo-drugs aims to solve these problems, as well as to adapt the new structures to modern therapies, personalized therapies and targeted molecular therapies. The design of new inorganic antitumor compounds aims to optimize their chemical design and pharmacological properties. Ideally, the new chemotherapeutic agent should be active in low doses, have minor adverse effects, have good bioavailability, high affinity for tumor cells, be able to be targeted to tumor cells without affecting healthy cells. In the same context, the development of efficient systems of transport and delivery to the target of chemotherapeutic drugs represents a major challenge for the scientific community [135,136].
  Currently, studies are focused on the design of delivery systems that allow the transport of a large number of platinum ions, protect the chemotherapeutic agent from premature degradation and inactivation processes, and allow its transport to the target while preventing interaction with healthy tissues. The indisputable advantages of using effective systems for transport and delivery to the target, macroscopic or nano-sized, are the possibility of drastically reducing the doses of chemotherapy administered and increasing the concentration of the bioactive agent accumulated in tumor cells and tissues [137,138].
  N-(2-hydroxypropyl)methacrylamide is one of the most commonly used polymers for conjugation with antineoplastic agents. In the case of AP5280, the chloride anions in cisplatin are replaced by a bidentate malonate anion in the terminal position in the N-(2-hydroxypropyl)methacrylamide structure [139]. Based on the results of preclinical studies, which show a 20-fold reduced toxicity compared to ciplatin and a clearly superior tissue accumulation, the compound was advanced to clinical trials [140]. The less promising results led to the abandonment of clinical trials in phase I, the developer company turning its attention to its analogue, AP5346 or ProLindac, in which the compound DACHPt, (1,2-diaminocyclohexane)Pt(II), is conjugated to the N-(2-hydroxypropyl)methacrylamide polymer via an amidomalonate group. The polymeric conjugate, stable at physiological pH, releases DACHPt at lower pH values from the hypoxic extracellular space of the neoplastic tissue [141]. The results of preclinical studies and of phase I clinical studies indicate an efficiency comparable to that of oxaliplatin on various tumor cell lines and in the treatment of solid tumors, good tolerance and reduced hematotoxicity. Phase I/II clinical studies that included patients with ovarian cancer in advanced stages, confirmed that the efficiency of the conjugate is similar to that of standard oxaliplatin. Administration of ProLindac was not associated with the onset of acute neurotoxicity, the main adverse effect that limits dose escalation in oxaliplatin treatment. In the case of a clinical trial in which ProLindac is used together with Paclitaxel in the combined therapy of advanced ovarian cancer, although the completion deadline has been exceeded, the results have not yet been made public [142].
  BP-C1 is a cisplatin derivative in which the chemotherapeutic agent is fixed by some polycarboxylic acid residues at the level of an amphiphilic polymer derived from lignin. The product has passed the stage of preclinical and phase I and II clinical studies on stage IV metastatic breast cancer and on inoperable metastatic pancreatic cancer. The results of monotherapy in breast cancer indicate an efficiency comparable to cisplatin and superior to carboplatin, as well as a lower toxicity compared to cisplatin. The treatment was associated with a low incidence of adverse effects and, as a result, with a significant improvement in the patients quality of life [143].
  Another type of polymeric nanoparticle, derivative of cisplatin, is NC-6004 or nanoplatin. Hydrophobic micellar particles functionalized with PEG are currently in phase I, II or III clinical trials, being evaluated in the treatment of different types of neoplasia: pancreatic cancer (phase III clinical trials), head and neck cancer (phase I clinical trials ), and other solid tumors (phase II clinical studies) [144]. The first results indicated a good tolerance to the treatment and a reduced incidence of adverse effects, with greatly reduced nephrotoxic effects compared to cisplatin. Nanoplatin is also included in other ongoing clinical trials evaluating combined therapy with 5-fluorouracil and cetuximab (phase II clinical trials), with pembrolizumab (phase II clinical trials) and with gemcitabine (phase II clinical trials phase I and II clinics) [145,146].
  The results of clinical studies demonstrate the important role that these transport and target delivery systems play especially in reducing the incidence and severity of adverse effects associated with therapy with platinum ion compounds. Although the reduction of toxicity brings enormous benefits in terms of the patients’ quality of life, the efficiency, which is often comparable to that of cisplatin, justifies the continuation of studies in order to identify new strategies that significantly improve clinical results.
  In the near future, advanced techniques of metalloproteomics, genomics and molecular biology will be able to fully elucidate the mechanism of action of metal complexes. Thus, the physico-chemical and structural properties of the coordinating compounds will be able to be anticipated in order to obtain effective antitumor agents [19].

## 5. Conclusions

The discovery of platinum drugs have a great impact on the clinical cancer chemotherapy in that they have been used to treat different malignancies including colorectal, non-small cell lung and genitourinary cancers. Unfortunately, the treatment efficacy of cisplatin was hindered by drug resistance and severe side effects. Recently researchers have been engaged in the discovery of the non platinum complexes, and mononuclear and multinuclear metallic complexes. The understanding of the mechanisms of action of an increasing number of metal complexes impressively demonstrates the broad variety of cell death pathways activated by metal complexes. Metal complexes that can activate alternative pathways of cell death highlight possible opportunities for the treatment of tumors resistant to the currently available drugs. Copper and ruthenium complexes probably have greater potential over platinum complexes, showing reduced toxicity, a new mechanism of action, selectivity for tumor cells and the posibility of non-cross-resistance. The current article briefly reviews the achievements concerning the anticancer activity of platinum, copper and ruthenium metal complexes, focusing the discussion on past, present and future aspects.
